# Autophagy-associated dengue vesicles promote viral transmission avoiding antibody neutralization

**DOI:** 10.1038/srep32243

**Published:** 2016-08-25

**Authors:** Yan-Wei Wu, Clément Mettling, Shang-Rung Wu, Chia-Yi Yu, Guey-Chuen Perng, Yee-Shin Lin, Yea-Lih Lin

**Affiliations:** 1 Institute of Basic Medical Sciences, College of Medicine, National Cheng Kung University, Tainan, Taiwan; 2 Department of Microbiology and Immunology, College of Medicine, National Cheng Kung University, Tainan, Taiwan; 3 Institute of Human Genetics, CNRS-UPR1142, Montpellier, France; 4 Institute of Oral Medicine, College of Medicine and Hospital, National Cheng Kung University, Tainan, Taiwan; 5 Center of Infectious Disease and Signaling Research, National Cheng Kung University, Tainan, Taiwan

## Abstract

One of the major defense mechanisms against virus spread *in vivo* is the blocking of viral infectibility by neutralizing antibodies. We describe here the identification of infectious autophagy-associated dengue vesicles released from infected cells. These vesicles contain viral proteins E, NS1, prM/M, and viral RNA, as well as host lipid droplets and LC3-II, an autophagy marker. The viral RNA can be protected within the autophagic organelles since anti-dengue neutralizing antibodies do not have an effect on the vesicle-mediated transmission that is able to initiate a new round of infection in target cells. Importantly, such infectious vesicles were also detected in a patient serum. Our study suggests that autophagy machinery plays a new role in dengue virus transmission. This discovery explains the inefficiency of neutralizing antibody upon dengue infection as a potential immune evasion mechanism *in vivo*.

Dengue is one of the most important arthropod-borne viral diseases in humans globally. It is estimated that approximately 390 million infections occur every year, of which 96 million cases show apparent clinical manifestations^1^, including severe dengue hemorrhagic fever and shock^2^,^3^,^4^. The actual mechanisms leading to the pathogenic causes of severe dengue remains at large in spite of many hypotheses have been proposed^5^. Currently, there is no anti-viral modality available, palliative care is the current standard practice to affected patients.

Dengue virus (DENV) belongs to the family *Flaviviridae* of genus *Flavivirus.* Its genome contains single positive-stranded RNA and encodes three structural proteins including capsid protein (C), premembrane/membrane protein (prM/M) and envelope protein (E) as well as seven nonstructural proteins (NS1 to NS5), responsible for numerous functions including viral RNA replication and protein synthesis. There are four serotypes of DENV, and each serotype by itself is capable of inducing the wide spectrum of dengue diseases. The E protein interacts with several receptors for DENV entry and attachment^6^,^7^,^8^,^9^, and is the major protein eliciting a serotype-specific antibody response in the infected host. Theoretically, neutralizing antibodies elicited by the same serotype virus are capable of inhibiting the subsequent infection by the same serotype^10^, but recently, it has been demonstrated that this may not be the case^11^. In addition, the limited cross-reactivity of neutralizing antibodies may result in detrimental outcomes – amplification of DENV infection and induction of severe diseases^11^,^12^,^13^,^14^,^15^. Why there is a limited capacity for neutralizing antibody to DENV remains unknown.

The cell-to-cell transmission has been suggested to be one of causes since this helps the virus to evade inhibitory effect by neutralizing antibodies and spread efficiently to adjacent cells. For instance, human immunodeficiency virus type 1 (HIV-1) utilizes virological synapses and tunneling nanotubes for transmission^16^,^17^, assisting the virus to escape potent neutralizing antibodies^18^. Hepatitis C virus (HCV) has been reported to infect human hepatoma cell line via cell-to-cell transmission^19^, eschewing from neutralizing antibodies^20^ by packaging virions in exosomes^21^. Despite both HCV and DENV belong to the same virus family; upregulation of exosomes has a negative effect on DENV^21^. Hence, with the ineffective pre-existing antibodies in dengue patients, it is speculated that DENV might use an alternative viral morphology^22^ or transmission pathway to avoid neutralizing antibodies.

Autophagy is a highly conserved cellular metabolic pathway by degradation of intracellular damaged organelles or proteins^23^, and is an anti-bacteria^24^ and anti-viral^25^ defense system in eukaryotic cells. Autophagosome is a double-membrane structure forming during the autophagic flux^26^, a process involves the expression of autophagy-related genes (Atg)^27^ and the combination between phosphatidylethanolamine (PE) and microtubule-associated protein 1 light chain 3 (LC3)/Atg8^28^. The functionality of autophagy in DENV infection appears to be cell type dependent; an inhibitory effect in monocytic cells^29^, while an enhancement of DENV output in Huh7 cells^30^. Metabolically, DENV can utilize the autophagy to degrade lipids to gain energy for the replication^31^. Interestingly, unconventional secretion pathway through autophagy has been reported to participate in exocytosis, which facilitates pathogens divert the autophagy process to help their survival by replicating on the membrane structure of autophagosome^32^. Furthermore, recent reports suggest that autophagy also participates in the extracellular delivery of a number of cytosolic proteins from the cytosol^33^,^34^,^35^. We, therefore, address the question whether autophagy may provide a platform not only for DENV replication but also assisting in the transmission of DENV.

## Results

### Close-contact co-culture enhances DENV infection rate

**Figure 1:**
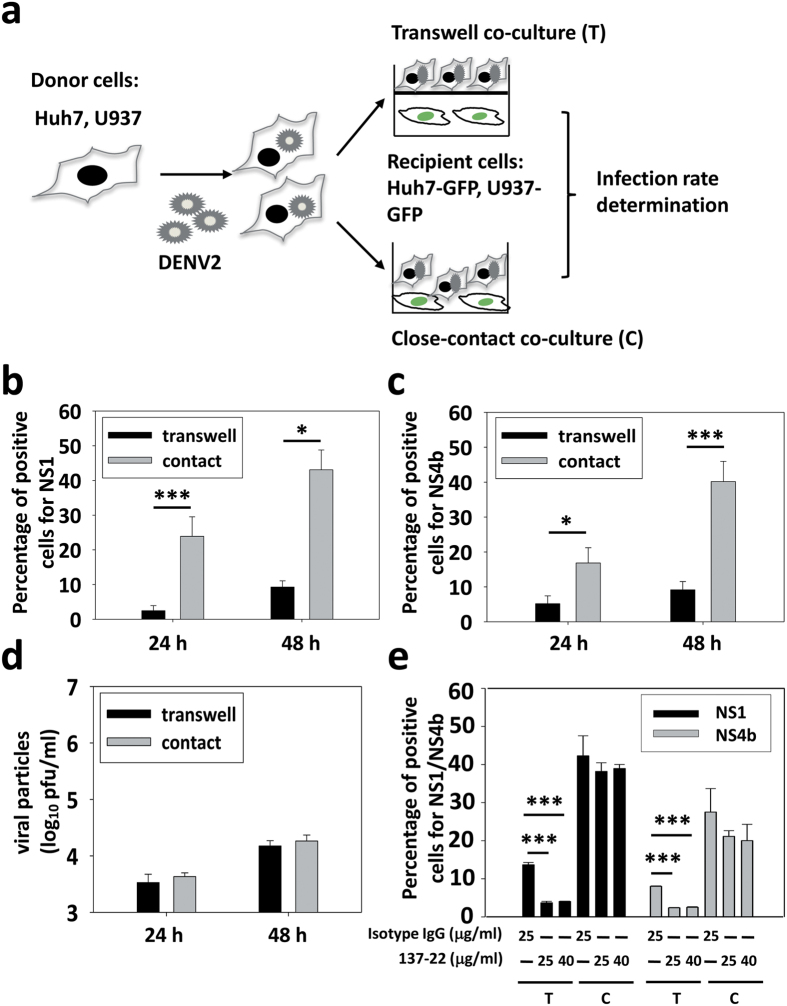
Efficient infection of DENV avoids neutralizing antibody in a direct-contact co-culture. **(a)** Schematic illustration of the experimental procedure. (**b,c**) DENV2 (PL046)-infected Huh7 cells were used as donors to further infect Huh7-GFP cells harboring an integrated *GFP* gene. Infected Huh7 cells were either cultured in transwells on top of the recipient cells or directly with Huh7-GFP cells for 24 h. The infection rate was quantified using a specific antibody against dengue NS1 **(b)** or NS4b **(c)** and flow cytometry analysis. **(d)** Comparable virion production in transwell and close-contact co-culture conditions. DENV2-infected Huh7 cells were either cultured in transwells on top of the recipient cells or directly with Huh7-GFP cells for 24 h. The virion production in the culture media was quantified using plaque assay. **(e)** Two different concentrations of neutralizing antibody were added in transwell and close-contact co-culture during the infection. The infection rate of the recipient was detected by flow cytometry using NS1 or NS4b antibodies. Data represent the mean ± SD of three independent experiments, *P < 0.05; ***P < 0.001; paired *t*-test.

To mimic a free virion-mediated or a cell-to-cell transmission condition, a schematic drawing was outlined to approach the purpose (Fig. 1a). Briefly, we used T-clear transwells with the pore size of mesh at 3 μm or close-contact co-culture between DENV-infected donor cells (MOI = 5) and recipient cells overexpressing GFP. Recipient cells were seeded in the lower chamber overnight and then DENV-infected donor cells were added to the apical chamber (transwell) or donor cells were directly added to recipient cells (close-contact), and the infection rate was analyzed by FACS at indicated times. The permeability of the membrane of transwell to DENV was verified by the infection of recipient cells resulting from the free DENV2 virions added in the upper chamber (Supplementary Fig. S1). Experimental results from three independent studies revealed that the infection rate in human hepatoma recipient cells in close-contact co-culture was much higher than in transwell culture at 24 and 48 h post-infection, as assayed by NS1 (Fig. 1b) or NS4b (Fig. 1c) expression, respectively. The difference in infection rate between transwell and close-contact co-culture assays was not due to a reduced virion production in transwell culture, since the number of free virions in the culture media was similar in both conditions at indicated time points (Fig. 1d). In addition, a similar phenomenon was observed in a monocytic cell line U937 (Supplementary Fig. S2), suggesting that higher infection rate in close-contact co-culture may be mediated by a much more efficient transmission mechanism than free virions.

To block the infection of virions, neutralizing antibody was added in both transwell and close-contact co-culture. A neutralizing antibody (137-22)^36^ was added to the culture media in the lower chamber before seeding the apical chamber. This antibody at the concentration of 25 μg/ml efficiently blocked DENV infection in Huh7 cells (Supplementary Fig. S3). The blocking effect could be observed in the transwell system, but failed to mitigate the infection in the close-contact co-culture setting with the 173-22 neutralizing antibody at the experimental concentration (Fig. 1e).

### Autophagy-deficient donors decrease transmission rate in close-contact co-culture

**Figure 2:**
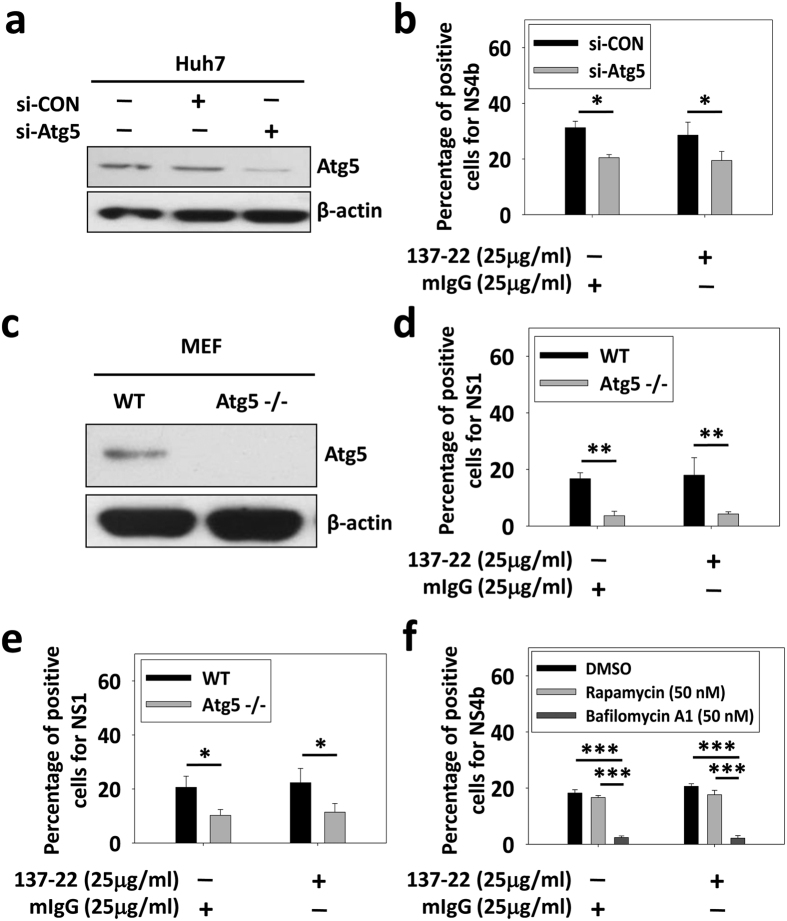
Autophagy-deficient donor cells decrease the infection rate of recipients in close-contact co-culture condition. **(a)** Huh7 cells were transfected with control or specific siRNA against Atg5. The expression level of Atg5 was quantified by Western blotting. **(b)** Control (MOI = 1) and knockdown cells (MOI = 5) were infected with DENV2 and close-contact co-cultured with Huh7-GFP cells with the isotype control or neutralizing antibody 137-22 for 48 h. The infection rate of the recipient was detected by flow cytometry with an NS4b monoclonal antibody. **(c)** The expression levels of Atg5 in wild-type and *Atg5*^−/−^ MEF (mouse embryonic fibroblast) were quantified by Western blotting. **(d)** DENV2-infected wild-type or *Atg5*^−/−^ MEF with MOI = 5 were close-contact co-cultured with Huh7-GFP cells with the isotype control or neutralizing antibody 137-22 for 48 h. Dengue infection rate of the recipient was detected by flow cytometry with an NS1 monoclonal antibody. **(e)** DENV2-infected wild-type (MOI = 5) or *Atg5*^−/−^ MEF (MOI = 10) were close-contact co-cultured with Huh7-GFP cells with the isotype control or neutralizing antibody 137-22 for 48 h. Infection rate of the recipient was detected by flow cytometry with an NS1 monoclonal antibody. **(f)** DENV2-infected Huh7 cells were co-cultured with Huh7-GFP cells for 24 h with rapamycin or bafilomycin A1. The infection rate was quantified using a specific antibody against dengue NS4b and flow cytometry analysis. Data represent the mean ± SD of three independent experiments, *P < 0.05; **P < 0.01; ***P < 0.001; paired *t*-test.

The autophagosome has been reported to facilitate protein secretion bypassing classical protein secretion pathways, such as endoplasmic reticulum (ER) and Golgi apparatus^37^. We, therefore, investigated the role of autophagy in DENV replication and in its transmission. The essential protein, Atg5, in autophagy was depleted by RNA interference in Huh7 cells (Fig. 2a). The Atg5-depleted Huh7 cells were infected with DENV at an MOI of 5, which were then co-cultured with Huh7-GFP cells. There was a significant decrease in the infection rate of Huh7-GFP cells that were close-contact co-cultured with DENV-infected Atg5-siRNA donor (Fig. 2b). Furthermore, the 137-22 neutralizing antibody did not alter the transmission pattern (Fig. 2b). To eliminate the effect of residual Atg5 in the siRNA-treated cells, Atg5^−/−^ mouse embryonic fibroblasts (MEF) (Fig. 2c) were infected with DENV and utilized as donors to infect Huh7-GFP cells for the close-contact co-culture assay. Results showed that the infection rate was dropped significantly in the autophagy-deficient Atg5^−/−^ MEF compared to the wild-type MEF (Fig. 2d). In order to overcome the decreased DENV replication due to autophagy deficiency in si-Atg5 Huh7 cells and Atg5^−/−^ MEF donors, similar virus productions were obtained with higher MOI of DENV to infect these cells for the close-contact co-culture assay (Supplementary Figs S4 and S5). To our surprise, the infection rate in autophagy-deficient cells decreased by 50% compared to wild-type MEF (Fig. 2e). Again, the addition of neutralizing antibody had no effect on transmission rate in close-contact co-culture setting (Fig. 2e). Since Atg9 vesicle is an important component of autophagy initiation^38^, the study was performed with Atg9 knockdown donors as well. Results showed that the similar reduction effect to Atg5 knockdown donors was observed in Atg9 knockdown donors (data not shown), suggesting the decreased infection rates are not due to the lower virus production of autophagy-deficient donor cells. Known drugs that are capable of causing effects, either inhibitory (bafilomycin A1) or enhancement (rapamycin), were used to treat Huh7 donor cells and subsequently infected with DENV. The infection rate in bafilomycin A1 treated donor cells was decreased dramatically while rapamycin treated donor cells did not have an effect (Fig. 2f). These results demonstrate that autophagy is involved in this novel dengue close-contact transmission.

### Extracellular vesicles contain DENV proteins and autophagy marker LC3

**Figure 3:**
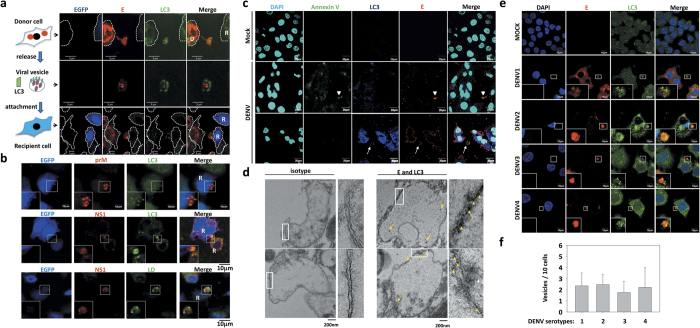
Dengue viral vesicles contain autophagy machinery. **(a)** After DENV2 infection for 24 h (MOI = 5), Huh7 cells were close-contact co-cultured 12 h with Huh7-GFP cells. Cells were fixed, stained with anti-E and -LC3 antibodies and analyzed with confocal microscopy. Upper line emphasizes a donor cell field, line 2 a vesicle field and bottom line a recipient cell field. Donor cells (D); Recipient cells (R); Cell edge (dashed line). **(b)** The presence of other viral proteins, prM, NS1 and lipid droplet (LD) was also detected using specific antibodies and lipid droplet dye. R: recipient cells. **(c)** After DENV2 infection for 48 h (MOI = 5), Huh7 cells were fixed and stained with anti-dengue E, LC3, and annexin V antibodies and analyzed with confocal microscopy. Apoptotic body (arrow head); Vesicle (arrow bar). **(d)** The vesicles were detected by TEM, and LC3 and E were detected by 10 nm (arrow head) and 20 nm (arrow bar) immunogold, respectively. **(e)** Four serotypes of DENV produced by C6/36 cells were used to infect Huh7 cells. The presence of E and LC3 proteins in secreted viral vesicles was detected using confocal microscopy. **(f)** The number of vesicles was quantified from (e). Data represent the mean ± SD of three independent experiments; paired *t*-test.

Verification of the involvement of autophagy in DENV transmission, DENV E antigen and LC3, an autophagy marker, were double stained in the close-contact co-culture system, and images were captured by confocal microscopy. Schematic showing the sequence of the autophagy machinery involved in DENV transmission and the likely corresponding confocal images were demonstrated in Fig. 3a. The involvement of autophagy in DENV-infected donor cells was readily observed (Fig. 3a, upper panel). In addition, autophagy marker containing DENV viral vesicle apparently secreted from donor cells at 12 h post-infection was captured (Fig. 3a, middle panel), which subsequently infected the recipient cell (Fig. 3a, bottom panel). Vesicles with the size at 2–5 μm in width, slightly larger than classical autophagosomes (0.5–1.5 μm) harboring other viral proteins such as prM, NS1, and host related lipid droplet were detected in these dengue vesicles as well (Fig. 3b). The dengue vesicles apparently did not associate with annexin V (Fig. 3c middle panel, arrow head indicating apoptotic body vs. bottom panel, arrow bar indicating vesicle), suggesting these vesicles were not cellular debris or apoptotic structures. The dengue vesicles were subjected to immunogold labeling and visualized under transmission electron microscopy; images revealed that the viral E antigen was packaged by a double layer of membrane structure staining positive for autophagy LC3 marker (Fig. 3d). Furthermore, all four DENV serotypes were capable of producing these vesicles in infected donors cells efficiently (Fig. 3e) and equally (Fig. 3f). Results suggest that the production of autophagy-associated dengue vesicles in infected cells is a general phenomenon in DENV infection.

### Reduction of dengue vesicles in the DENV-infected autophagy-deficient cells

**Figure 4:**
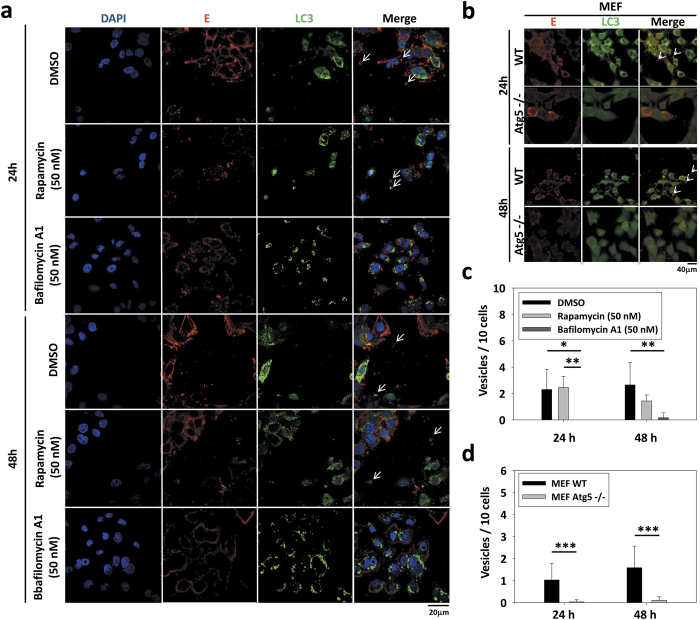
The number of dengue vesicles is reduced in the autophagy-deficient cells. **(a)** DENV2-infected Huh7 cells at an MOI of 1 were treated with rapamycin or bafilomycin A1. After 24 and 48 h of incubation, cells were fixed and stained with anti-E and -LC3 antibodies and analyzed with confocal microscopy. **(b)** DENV2-infected wild-type (MOI = 5) or *Atg5*^−/−^ MEF (MOI = 10) were close-contact co-cultured with Huh7-GFP cells for 24 or 48 h. Cells were fixed, stained with anti-dengue E and LC3 antibodies and analyzed with confocal microscopy. Vesicle (arrow bar). (**c**,**d**) The numbers of vesicles from **(a,b)** were quantified and represent the mean ± SD of ten figures in each groups, *P < 0.05; **P < 0.01; ***P < 0.001; paired *t*-test.

**Figure 5:**
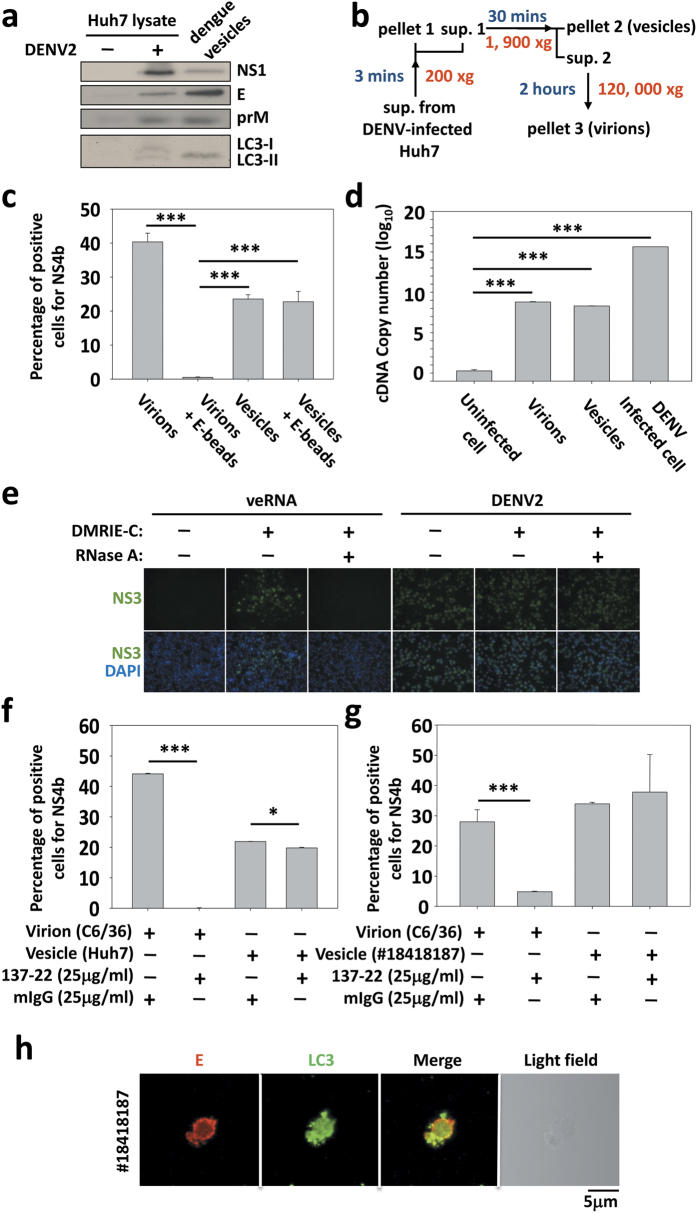
Dengue vesicles containing viral RNA are infectious. **(a)** Huh7 cells were infected by DENV2 at an MOI of 1, and the supernatant was harvested 48 h post-infection to obtain the vesicles. The E, prM, NS1 and LC3 expression levels were detected by Western blotting. **(b)** Schematic illustration of vesicle isolation. **(c)** Virions-depleted vesicles by anti-E-coated magnetic beads. Isolated virions and vesicles were incubated with anti-E-coated magnetic beads overnight and retreated to naïve Huh7 cells. After 48 h incubation, infection rates were detected by NS4b. **(d)** Total RNA was extracted from virions of C6/36, and vesicles from infected or non-infected Huh7. The presence of viral RNA was detected by qRT-PCR using specific primers pairing NS1 sequence. **(e)** BHK cells were infected or transfected (DMRIE-C reagent) by DENV2 or veRNA (RNA from purified vesicles) with or without RNase A treatment. After 24 or 72 h, the expression of viral NS3 was revealed by IFA. **(f)** Huh7 cells were infected with vesicles or virions isolated from the culture medium of infected cells. The infection rate in the presence or absence of neutralizing antibody 137-22 was quantified using an antibody against NS4b and flow cytometry analysis. **(g)** Huh7 cells were incubated with vesicles separated from a dengue patient (#18418187) or virions isolated from C6/36 cells in the absence or presence of the neutralizing antibody (137-22). The infection rate was quantified using an antibody against NS4b and flow cytometry analysis. **(h)** Dengue vesicles were isolated from serum of a dengue patient (#18418187) by centrifugation as described above. The sample was analyzed using anti-dengue E and LC3 antibodies and confocal microscopy. Data show one representative experiment out of 3, the mean ± SD of triplicates. *P < 0.05, ***P < 0.001; paired *t*-test.

Aforementioned on autophagy-associated dengue vesicles had been captured by confocal microscopy. To further confirm the involvement of autophagy machinery in production of dengue vesicles, cells were pretreated with autophagy inhibitor bafilomycin A1 and enhancer rapamycin and infected with DENV. The dengue vesicles were visualized by confocal microscopy and quantified after 24 and 48 h infection. Data showed that even though production of dengue vesicles in these pretreated cells, the numbers of dengue vesicles were reduced in cells pretreated with bafilomycin A1, while unchanged was seen in rapamycin pretreated cells (Fig. 4a,c). The dengue vesicles released from cells pretreated with rapamycin apparently did not alter the levels and distribution of viral E and LC3 under the visualization of confocal microscopy (Supplementary Fig. S6). In addition, the number of dengue vesicles dramatically decreased in Atg5^−/−^ MEF as compared with wild-type MEF (Fig. 4b,d). Further analysis revealed that the dengue vesicles contained LC3-II (Fig. 5a), a unique molecule expressed on autophagosome. The cumulative data imply that the dengue vesicles are a secretion structure rather than cytosolic-containing cellular debris and that these autophagy-associated dengue vesicles may contribute to the close-contact transmission avoiding antibody neutralization.

### Infectious autophagy-associated dengue vesicles can escape antibody neutralization

The biological properties of the dengue vesicles were further characterized by isolation of the dengue vesicles and virions with house-developed protocol (Fig. 5b). As a control, specific neutralizing antibody 137-22 coated with magnetic beads was added to supernatant prior to isolate the dengue vesicles and virions. Results showed that the dengue vesicles were infectious and were unable to be neutralized by 137-22 antibody; in contrast, virions were readily neutralized by the antibody (Fig. 5c). There were estimated at 2 × 10^8^ copies of viral RNA in dengue vesicles derived from 2.88 × 10^9^ DENV-infected Huh7 cells after quantified by qRT-PCR of the NS1 sequence (Fig. 5d). To demonstrate the RNA within the vesicles is infectious and complete, RNA was extracted from isolated vesicles then pretreated with RNase prior to infect BHK cells. The infectivity was evaluated after the 72 h infection by IFA. Results showed that infectivity of viral RNA was derived from within the vesicles (Fig. 5e). Cumulative results suggest that the RNA-containing in vesicles is complete and infectious.

To verify that these dengue vesicles were able to escape neutralization by antibodies, we infected Huh7 cells with either free virions produced by C6/36 cells or purified dengue vesicles produced by Huh7 cells. Despite lower percentage of positive cells for NS4b in recipient Huh7 cells, dengue vesicles were still able to infect cells compared to the free virions in the presence of neutralizing antibodies (Fig. 5f). The physiological existence of the dengue vesicles in dengue patients was performed with isolated dengue vesicles from a patient serum. Patient-derived dengue vesicles were also infectious in recipient Huh7 cells and the infectivity of patient-derived dengue vesicles was not attenuated by the neutralizing antibody (Fig. 5g). Importantly, dengue vesicles isolated from patient sera presented the same markers, dengue E and LC3, as the Huh7-derived dengue vesicles described above (Fig. 5h).

## Discussion

Autophagy has been initially identified as a cellular defense mechanism to clear the invading agents; however, a growing body of evidence reveals the hijacking of host machinery by viruses for their replication and transmission. Pharmacological or gene silencing approach of autophagy pathway *in vitro* demonstrated an inhibition of growth and/or spread of these viruses. Multiple mechanisms have been suggested to be involved in the pro-viral function of autophagy, either direct effects on viral replication or indirect influences on the host immune and nonimmune-related activities, including serving as viral replication sites, promoting viral entry/uncoating and maturation, and suppressing innate anti-viral immunity. These processes regulate cellular metabolism and prevent premature cell death and degradation of intracellular anti-viral factors^39^. In line with our observation, various studies identified the secreted exosome harboring viral genetic materials in the transmission of hepatitis C virus and picornavirus^40^,^41^. However, the exact role of autophagy in DENV life cycle is not yet clearly understood.

Some studies had linked the induction of autophagy with viral replication, showing the co-localization of autophagy markers with viral replication markers^30^,^42^,^43^. Another study showed that LC3-I and EDEM1-containing membranes can serve as Japanese encephalitis virus replication but not LC3-II^44^, suggesting that flavivirus might replicate without autophagosome as a platform. Similar to other positive-stranded RNA viruses, DENV recruits host membrane to assemble its replication complex as part of a large organelle-like structure. The nature of the recruited membrane, such as ER and mitochondria, varies among different viruses. Others have suggested that replication does not co-localize with autophagy markers^31^ since replication of virus may not occur in autophagosome but rather viral replication itself causes ER stress resulting in autophagy induction^45^. Autophagy may then help to change the lipid composition of the organelles and lipid degradation can be used to gain energy for replication^31^. In addition to the *in vitro* studies, a report using a mouse model confirmed the induction of autophagy as shown by the increased LC3-II expression, double-membrane vesicles, and amphisome, which plays a promoting role in DENV replication^46^. However, another study argued that instead of an effect of autophagy in viral spreading, spautin-1, an inhibitor for deubiquitination of USP10 and USP13, inhibits the initiation of autophagosome and infectious particles in BHK cells^47^. This inhibition cannot be rescued by lipid complementation, suggesting that autophagy can be involved in maturation of dengue virions rather than RNA replication. A recent report showed that cell-to-cell spreading can occur for mosquito cells via vesicles that interact with the cellular protein tetraspanin C189 to avoid antiserum^48^. However, the described vesicles are much smaller and contain densely packed virions. The vesicles transmitted between human cells and in patient’s serum that we described here are 10 times bigger, and capsid protein was absent in the vesicle fraction (data not shown). This suggests that the virus does not need to produce a mature virion to transmit infection; we propose that viral RNA linked to a membrane structure might be enough.

Bafilomycin A1 is a V-ATPase inhibitor that blocks the late steps of autophagy through the inhibition of lysosome acidification. Besides, the membrane contact between selected targets and the vacuolar membrane is considered to be essential for the functional V-ATPase^49^. Since bafilomycin A1 possessing the inhibitory effects of vesicle transmission and secretion, the acidification of intracellular vacuole is necessary to the vesicle secretion. On the other hand, rapamycin inhibits mTOR which negatively regulates the activation of autophagy. However, rapamycin had no effect on the enhancement of vesicle secretion. The reason is that although the autophagic flux is activated by rapamycin at an early time, the dengue replication is still at a low level. There are no enough dengue proteins to form the dengue vesicle. In addition, the induction of autophagy is not directly correlated to the enhancement of autophagy-related secretory pathway and the key regulator between them is still unclear. However, we could not exclude the possiblity that the concentration of rapamycin at 50 nM might not be effective to enhance autophagy.

In the present study, we identify infectious dengue vesicles containing autophagy machinery, which can escape neutralizing antibody. These vesicles (2–5 μm) can be as big as 1/64 of the cell volume, a size not previously described, which forbid them to cross the membrane of transwells with the pore size of 3 μm. It might be possible that the virus uses the autophagy process to create a huge secretion structure, which is not an apoptotic body but rather a cargo for infectious material. The detection of these autophagy-associated dengue vesicles in patient’s serum or *in vitro* cell culture and the proof that they are infectious strongly implies the importance of autophagy machinery not only in replication but also in viral transmission. This study sheds a new light on early observations that dengue patient serum contains vesicle-like particles and that classical viral particles could not be visualized by electron microscopy in highly infectious acute samples^22^. This special vesicle structure may help explain the inefficiency of the host immune response against DENV infection.

## Methods

### Cell culture and lentiviral transduction

*Aedes albopictus* cell line C6/36 and human hepatoma cell line Huh7 were both from Dr. Huan-Yao Lei’s lab and cultured in DMEM supplemented with 5% or 10% fetal bovine serum (FBS). U937 was purchased from ATCC and cultured in RPMI supplemented with 10% FBS. The siRNA clones obtained from the National RNAi Core Facility (Institute of Molecular Biology/Genomic Research Center, Academia Sinica, Taiwan) were transfected into target cells. The si-Atg5 (ID TRCN0000151963) sequence is CCTGAACAGAATCATCCTTAA. The control RNA (ID TRCN0000072243) sequence is CTTCGAAATGTCCGTTCGGTT. The transwells (T-clear, pore size 3 μm) were from Corning.

### DENV production

C6/36 cells were infected with DENV at an MOI of 0.1 and maintained in 2% FBS DMEM. The virus was harvested 96 h post-infection and the viral titer was quantified using plaque assay. All four DENV serotype strains (serotype 1/766733A, serotype 2/PL046, serotype 3/739079A, and serotype 4/H-241) were obtained from the Center for Disease Control in Taiwan.

### Antibodies and reagents

Dengue specific antibodies, anti-E (137-22, 185-10), anti-prM (155-49) and anti-NS1 (13-F4-G5), were produced by our laboratory^36^. Anti-NS3 and anti-NS4b antibodies were purchased from GeneTex Inc. Anti-Atg5 and anti-Atg7 were from Cell Signaling Technology Inc., and anti-LC3 was from MBL International. Anti-mouse and anti-rabbit gold-conjugated antibodies were purchased from Abcam. Annexin V was stained by Annexin V-FITC Apoptosis Detection Kit. Rapamycin was purchased from MBL International. Bafilomycin A1, proteinase K and BSA were purchased from Merck Millipore. Magnetic beads were purchased from Invitrogen by Life Technologies. RNase A and DMRIE-C transfection reagents were purchased from Thermo Fisher Scientific Inc.

### Patient’s serum

Samples of acute dengue patients were collected according to the protocol approved by the institutional review board of National Cheng Kung University Hospital (NCKUH) IRB #A-BR-101-156. Enrolled patients were people older than 15 years of age, who visited NCKUH and were diagnosed with acute dengue virus infection. Infections were confirmed by the laboratory standards set forth by Taiwan CDC and in accordance with the WHO diagnostic guidelines. The informed consent was obtained from all subjects.

### Flow cytometry

The cells were fixed by 2% paraformaldehyde in PBS for 15 min then permeabilized by 2% saponin in PBS. After incubation with primary antibodies for 1 h and secondary antibodies for 0.5 h, samples were analyzed by FACS Calibur.

### Isolation of dengue virions and vesicles

Huh7 cells were infected at an MOI of 5 for 2 h and washed. Supernatant of cells was harvested 48 h post-infection and centrifuged for 3 min at 200× g to get pellet 1 and supernatant 1. The supernatant 1 was then centrifuged for 30 min at 1,900× g to get pellet 2 and supernatant 2. The pellet 3 was collected from the supernatant 2 after 2 h of centrifugation at 120,000 × g. Vesicles and virions were enriched in pellet 2 and pellet 3, respectively.

### Immunofluorescence microscopy

Cells were seeded on cover slides at a density of 1 × 10^6^/well. They were fixed by 4% paraformaldehyde (PFA) and permeabilized with permeable buffer (1% FBS, 0.1% sodium azide, 0.1% saponin). Dengue viral proteins and autophagy machinery were detected using specific antibodies and fluorescence confocal microscopy (FV1200, Olympus).

### Transmission electron microscopy (TEM) and immunogold staining

Isolated vesicles were fixed by 2.5% glutaraldehyde for 1 h at 4 °C. The sample was treated with 10% H_2_O_2_ in PBS for 10 min at 37 °C. After PBS wash, the sample was treated with protease K for 10 min at 37 °C and then fixed in 4% paraformaldehyde for 15 min. The 2% BSA was used to block the sample, and then primary antibodies were incubated with sample for 1 h at 37 °C. Two sizes of immunogold conjugated antibodies, 10 nm and 20 nm, were used to detect the LC3 and E, respectively. The technology of JEM-1400 TEM was supported by Dr. Shang-Rung Wu.

### RT-PCR and qRT-PCR

Total RNA was isolated from DENV-infected cells by QIAamp Viral RNA kit (QIAGEN). The isolated RNA was reverse transcribed by PrimeScripTM RT Reagent Kit. Specific NS1 primers and Fast SYBR^®^ Green Master Mix were used to determine the cDNA copy number, using NS1 plasmid serial dilutions as a standard. Primer sequences in qRT-PCR include forward primer: 5′-GCCAAAGTCACACACTCTATGG-3′ and reverse primer: 5′-CCTGCTGTTTGTGTGTGATAGC-3′.

### Immunofluorescence assay (IFA)

BHK cells were seeded overnight then transfected with veRNA using 1.5 μl DMRIE-C reagent. After 72 h incubation, cells were fixed in 3.7% paraformaldehyde then permeabilized by 0.1% triton X-100. The dengue antigen was detected by anti-NS3 antibody followed by fluorescence-conjugated secondary antibody. For DENV2 infection control, BHK cells were infected at an MOI of 5 for 24 h.

## Additional Information

**How to cite this article**: Wu, Y.-W. *et al*. Autophagy-associated dengue vesicles promote viral transmission avoiding antibody neutralization. *Sci. Rep.* **6**, 32243; doi: 10.1038/srep32243 (2016).

## Supplementary Material

Supplementary Information
