# Supplementary material for: Autophagy-associated dengue vesicles promote viral transmission avoiding antibody neutralization
Source: Sci Rep. 2016 Aug 25;6:32243. doi: 10.1038/srep32243 (PMC4997566; doi:10.1038/srep32243)
Supplement: Supplementary Information [file srep32243-s1.pdf]

Supplementary information for

Autophagy-associated dengue vesicles promote viral transmission avoiding antibody neutralization

Yan-Wei Wu[1, 2], Clément Mettling[5], Shang-Rung Wu[3], Chia-Yi Yu[4], Guey-Chuen Perng[2, 4], Yee-Shin Lin[1, 2, 4*]& Yea-Lih Lin[5*]

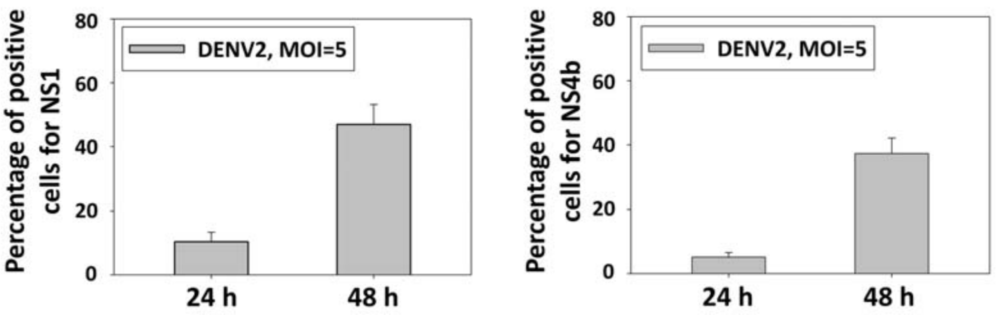

Supplementary Figure 1. Free virions can cross the transwell membrane. Free DENV2 (PL046) virions were put in the upper chamber of the transwell and infected (MOI = 5) Huh7-GFP cells harboring an integrated *GFP* gene in the lower chamber of the well for 24 or 48 h. The infection rate was quantified using a specific antibody against dengue NS1 or NS4b.

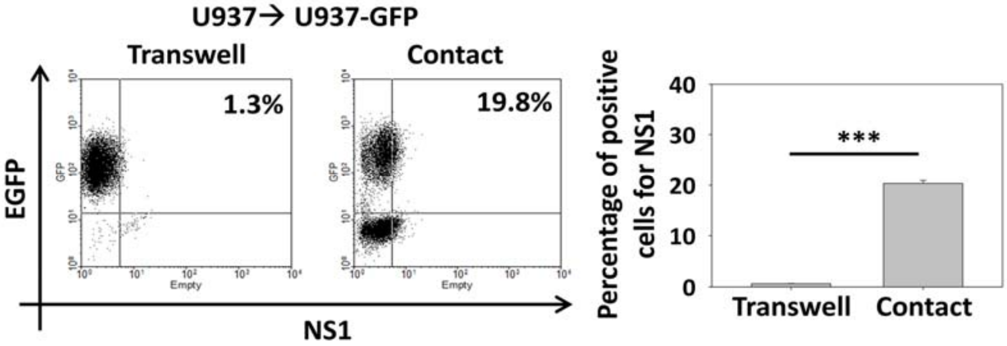

Supplementary Figure 2. Efficient transmission of dengue infection through close-contact co-culture. DENV2-infected U937 cells were either cultured in transwells on top of the recipient cells or close-contact with U937-GFP cells for 72 h. The infection rate of the recipient cells was quantified using an anti-NS1 antibody and flow cytometry analysis. ***, $P < 0.001$; paired *t*-test.

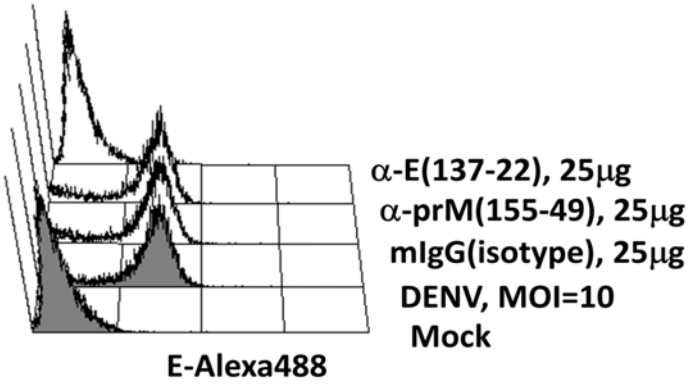

Supplementary Figure 3. Efficient inhibition of DENV2 infection by a neutralizing antibody. Huh7 cells were infected with DENV2 at an MOI of 10 in the presence of various and isotype control antibodies. The infection rate of Huh7 cells was quantified using another anti-E antibody and flow cytometry analysis.

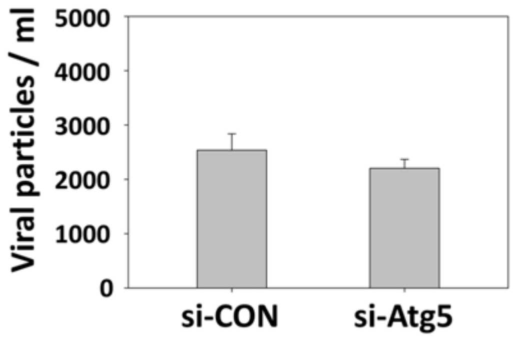

Supplementary Figure 4. Compensation of the DENV replication in autophagy deficient donors using Atg5 knockdown cells. The si-CON and si-Atg5 cells were infected by DENV2 at the MOI of 5 and 10, respectively. After 24 h, viral titers were quantified by plaque assay.

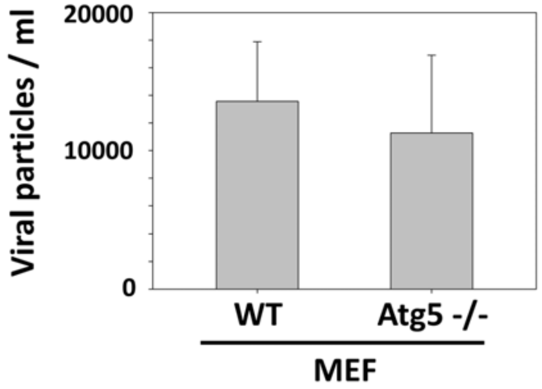

Supplementary Figure 5. Compensation of the DENV replication in autophagy deficient donors using Atg5 knockout cells. Wild type and *Atg5* $^{-/-}$ MEF cells were infected by DENV2 at the MOI of 2 and 10, respectively. After 24 h, viral titers were quantified by plaque assay.

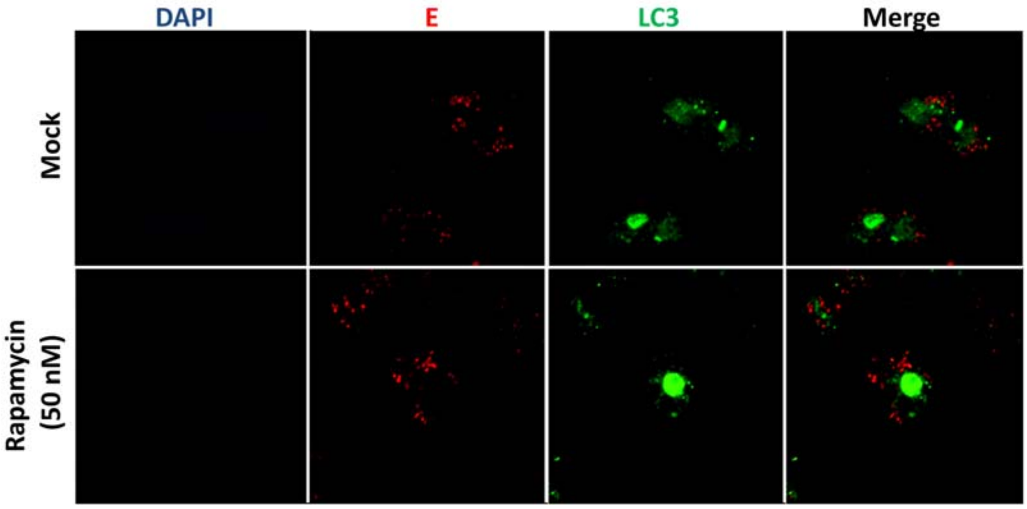

Supplementary Figure 6. Identical physiological characteristics of dengue vesicles with or without treatment of rapamycin. DENV2-infected Huh7 cells at an MOI of 1 were treated with rapamycin. After 24 and 48 h of incubation, cells were fixed and stained with anti-E and -LC3 antibodies and analyzed with confocal microscopy.
